# The Stake of Professional Reputation

**Published:** 1912-02-03

**Authors:** 


### The Hospital

The Workers' Newspaper of Administrative Medicine and Institutional Life, Administration, National Insurance and Health.

No. 1334, Vol. LI. SATURDAY, FEBRUARY 3rd, 1912. PRICE ONE PENNY.

### THE STAKE OF PROFESSIONAL REPUTATION.

According to what we know of the National Insurance Act, candidates for public medical employment are to be enrolled in panels for each district, amongst the members of which panels patients will have free choice. Very clearly, then, the layman's estimate of the comparative merit of different medical men remains a matter of great importance to the latter. It is, of course, true that the excellent principle of judgment by peers will obtain largely as regards the first half of the above procedure. Very wisely, the opinion of the profession itself upon its members is to be invoked to exclude notorious incompetence or misconduct. But one must hope that the bases of selection will be wide and liberal—that, for instance, the test of actual performance will not be outweighed by that of academic qualification. The prestige of this latter, among both lay and professional people, is quite high enough. Not long ago a certain Cabinet Minister, speaking at a certain ancient medical school in the north of these islands, imputed surpassing worth to its graduates, enlarging upon his theme so as to make it clear he spoke not merely in compliment. Now to those with inside knowledge of medical education this politician's remarks lowered his reputation. For, as a matter of fact, a pupil of this school, where students vastly outnumber patients, thinks himself lucky to get one of the smallest of minor operations to do; whereas at a certain London hospital, where matters find themselves more fortunately adjusted, he might (under proper supervision, of course) have several major ones for the asking. At the former institution, too, students are examined by their own teachers. Leaving quite out of count wild tales of favouritism to athletes and others, it will be conceded that the latter's plan of examination by strangers is preferable. In the University a strange practice was certainly lately in vogue whereby an unsuccessful candidate might, by expostulating tactfully with his late examiner, become a successful one after all; at the humbler seat of medical learning such behaviour would ensure an enforced respite from examination for nine months. Finally, it happens to be the case that the library at the northern seminary is without what is perhaps the leading German medical periodical; its librarian—courteous and obliging as librarians always are—recently confessed this fact with a blush. Now at Lincoln's Inn Fields the Royal College of Surgeons possesses, besides the famous museum, a medical library, which is not only far the finest medical library in Great Britain and Ireland, but one also hardly to be surpassed in the world. The relative success of northern and southern trained medical men in entering the existing public services goes to bear out the above painful comparison, the excuse for which must be a most influential man's ignorance. Not to go as far as that famous cockney, Mr. Jorrocks, who argued that sans exception the best of everything was to be found in London, one might still deprecate a little the half-educated person's reverence for the letters M.D.

How distrustful, on reflection, should a medical man become of his opinion of the professional merit of lawyers, soldiers, business men, parsons, engineers, artists, mechanics, and all specialists in knowledge, for what bad judges of physicians all these classes constantly show themselves to be! Not of surgeons, perhaps, for their work is more easily appraised than the more complex and more inexact medical therapeutics. In the latter the combination of a bold front, a gold watch, and a slightly unscrupulous exercise of worldly common sense will impose upon many. This common and obvious case has been enough insisted upon, yet professional success has often depended upon other attributes just as unessential. The sociable doctor strengthens his connection by allying himself with some definite division of the community, be it churchfolk or chapel, freemasons or freethinkers, socialists or clubmen. Probably he must just be simply following his own inclination in order to benefit much thereby, none the less this is one easily traceable factor in the growth of some large practices. Again, what is it that gives patients the impression of " cleverness "? The subject is as interesting to some doctors' wives as is, to most people, that larger one of the way in which certain usually envied people undoubtedly attract the opposite sex. Intellect is not by way of being overrated in this country, but yet the careful observer will have noted cases in which laudable admiration of an intellectual medical man involved the mistaken deduction that he was therefore a good doctor. The fallacy of this syllogism is not "undistributed middle," but the whole middle term itself—namely, that a good doctor is a clever man. A certain twelve years' experience of medicine has revealed that of the half-dozen of its exponents met with who owned really high power of intellect, only one had to his credit a real professional achievement, and he was a pathologist. The peculiarly and innately capable clinician, with or without a handle to his name, is an execrable judge of prose style, and does not go to picture galleries for his holiday. He is under the impression that politicians, not thinkers, are the leaders of progress, and would heartily dislike Bacon's "full man," the one who might tell him, in all innocence and almost in one breath, the birth-rate of ancient Rome and the first-class fare to the Balearic Islands; instead of talking sensibly about the main items of the newspaper placards. And this is just as it should be, for in the actual workaday business of treatment brains of fine quality are a hindrance. The moral of it all is that in placing medical men the greatest weight should be given to actual performance, for clinical talent, like talent of every other kind, is a highly complex mixture of different faculties, and the only proof of its presence is practical. Those who have given this proof do not deserve to suffer in any reconstitution of the profession. If they should so suffer, then the rest of the community would suffer too.

